# Applying Support Vector Machines for Gene ontology based gene function prediction

**DOI:** 10.1186/1471-2105-5-116

**Published:** 2004-08-26

**Authors:** Arunachalam Vinayagam, Rainer König, Jutta Moormann, Falk Schubert, Roland Eils, Karl-Heinz Glatting, Sándor Suhai

**Affiliations:** 1 Department of Molecular Biophysics, Deutsches Krebsforschungszentrum (DKFZ), TP3, Im Neuenheimer Feld 580, Heidelberg, D-69120, Germany; 2 Theoretical Bioinformatics, Deutsches Krebsforschungszentrum (DKFZ), TP3, Im Neuenheimer Feld 580, Heidelberg, D-69120, Germany; 3 Institut für Medizinische Biometrie, Epidemiologie und Informatik (IMBEI), Johannes Gutenberg-Universität Mainz, 55101, Mainz, Germany

## Abstract

**Background:**

The current progress in sequencing projects calls for rapid, reliable and accurate function assignments of gene products. A variety of methods has been designed to annotate sequences on a large scale. However, these methods can either only be applied for specific subsets, or their results are not formalised, or they do not provide precise confidence estimates for their predictions.

**Results:**

We have developed a large-scale annotation system that tackles all of these shortcomings. In our approach, annotation was provided through Gene Ontology terms by applying multiple Support Vector Machines (SVM) for the classification of correct and false predictions. The general performance of the system was benchmarked with a large dataset. An organism-wise cross-validation was performed to define confidence estimates, resulting in an average precision of 80% for 74% of all test sequences. The validation results show that the prediction performance was organism-independent and could reproduce the annotation of other automated systems as well as high-quality manual annotations. We applied our trained classification system to *Xenopus laevis *sequences, yielding functional annotation for more than half of the known expressed genome. Compared to the currently available annotation, we provided more than twice the number of contigs with good quality annotation, and additionally we assigned a confidence value to each predicted GO term.

**Conclusions:**

We present a complete automated annotation system that overcomes many of the usual problems by applying a controlled vocabulary of Gene Ontology and an established classification method on large and well-described sequence data sets. In a case study, the function for *Xenopus laevis *contig sequences was predicted and the results are publicly available at .

## Background

Ongoing genome sequencing and recent developments in cDNA sequencing projects have led to an exponential rise in the amount of sequence information. This has increased the need for acquiring knowledge from sequences as to their biological function. Annotating a single sequence is the gateway to interpreting its biological relevance. However, the usefulness of these annotations is highly correlated with their quality. *Accurate *annotation has traditionally been maintained manually with the experience of individual experts and the experimental characterisation of sequences. However, the increasing gap between the amount of sequence data available and the time needed for their experimental characterisation demands computational function prediction in complementing manual curation [1-4]. Commonly, computational functional assignment is based on homologues identified from database searches [5]. Such an automated annotation process provides comparable results due to a uniform analysis of all query sequences across the same databases and the possibility of repeating the annotation to updated sequence data [6]. However, crucial aspects for consideration in automated annotation are i) the problems associated with the databases themselves: sequence errors, erroneous annotation due to spelling ambiguities, incomplete functional annotation, inconsistent functional annotation across databases, consistent but wrong annotation across databases, and ii) the problems associated with the inference, i.e. false positives, where an assignment is made on the basis of a wrongly inferred homology [3,7,8]. A number of excellent annotation systems have been developed to tackle these problems, e.g. RiceGAAS [9], GAIA [10], Genotator [11], Magpie [12], GeneQuiz [6], GeneAtlas [13] and PEDANT [14]. However, little has been done to quantify the annotation accuracy by defined benchmarks *and *establish a method to provide a confidence value for each annotation.

The current annotation, written in a rich, non-formalised language also complicates this automated process. We addressed this problem by applying a controlled vocabulary from Gene Ontology (GO) [15-17]. GO provides consistent descriptions of gene products in a species-independent manner. The GO terms are organised in structured, controlled vocabularies (ontologies) to describe gene products in terms of their associated biological processes, cellular components and molecular functions. An increasing number of GO-mapped sequence databases make it possible to replace traditional database searches with GO-related searches. These include databases such as GenBank [18], SWISS-PROT [18], SwissPROT/TrEMBL [19], the TIGR Gene Index [20] and several other genome databases. Many annotation approaches have now been developed based on Gene Ontology. The uncharacterised sequences are searched across GO-mapped protein databases and assigned with GO terms of the best hits [21,22]. Jensen and co-workers used neural networks to predict specific subsets of GO terms [23]. Furthermore, Schung *et al *predicted GO terms by intersecting domain profiles [24]. The SwissPROT/TrEMBL entries were associated with GO terms by an automated process coupled with manual verification [19]. Text mining and similarity searches were combined to annotate SWISS-PROT and GenBank entries with GO terms [18]. However, these approaches were either applied to specific GO subsets or did not provide defined benchmarks and confidence values for their predictions.

We have developed an automated system for large-scale cDNA function assignment, designed and optimised to achieve a high-level of prediction accuracy without any manual refinement. Our system assigns molecular function GO terms to uncharacterised cDNA sequences and defines a confidence value for each prediction. The cDNA sequences were searched against GO-mapped protein databases and the GO terms were extracted from the homologues. In the training phase, these GO terms were compared to the GO annotation of the query sequences and labelled correspondingly. We applied Support Vector Machines (SVMs) as the machine learning method to classify whether the extracted GO terms were appropriate to the cDNA sequence or not. In order to classify the GO terms we used a broad variety of elaborated features (attributes) including sequence similarity measures, GO term frequency, GO term relationships between homologues, annotation quality of the homologues, and the level of annotation within the GO hierarchy. To enhance the reliability of the prediction, we used multiple SVMs for classification and applied a committee approach to combine the results with a voting scheme [25]. The confidence values for the predicted GO terms were assigned based on the number of votes i.e. number of SVMs predicting particular GO term as correct. The performance of the system was benchmarked with 36,771 GO-annotated cDNA sequences derived from 13 organisms. It achieved 80% precision for 74% of the test sequences. We applied our annotation system to predict the function for *Xenopus laevis*, a widely studied model organism in developmental biology. Because many researchers are now focussing on the functional genomics of this organism, a demand exists for a quality annotation [26]. Therefore we applied our system to improve the quality and coverage of the existing annotation. We predicted the function for 17,804 *Xenopus laevis *contig sequences (from TIGR Gene Indices) yielding annotation with good confidence values for more than half of these sequences.

## Results

### General workflow of training and classification

The classifier (SVM) needs to specify attribute values (features) for a broad list of samples and a class label for each of these samples. Through the training samples it learns the feature patterns and tries to group them according to their class labels. After training, the algorithm assigns class labels to new samples according to the class that they best match.

**Figure 1:**
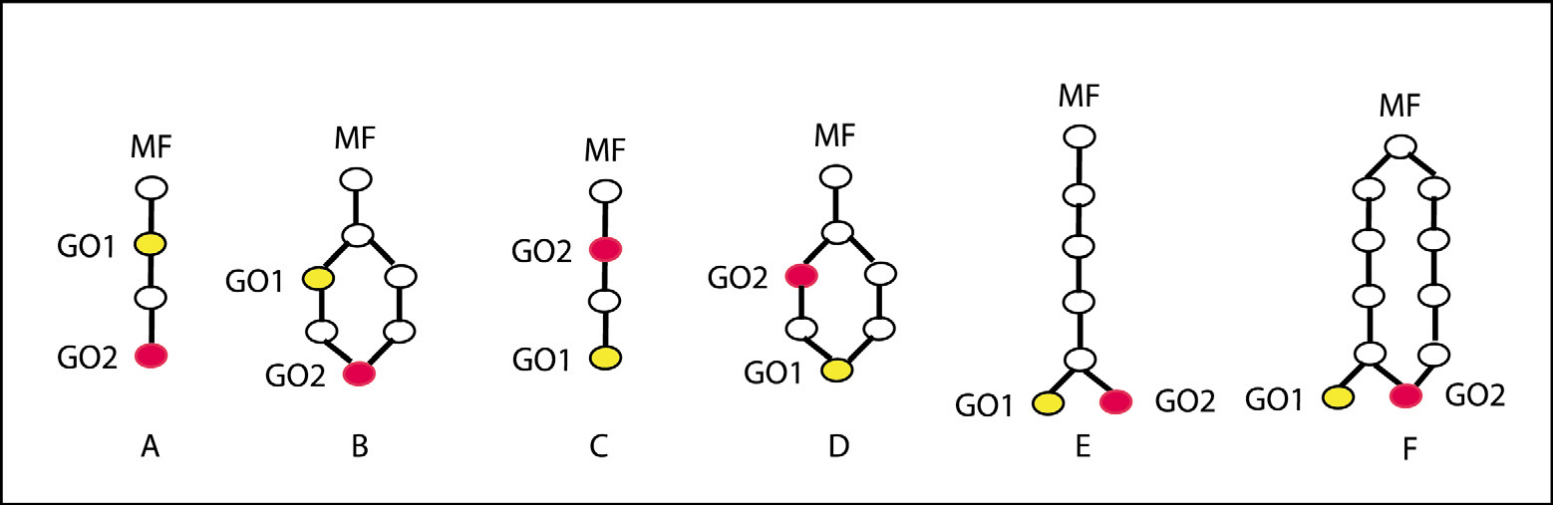
A schematic representation of possible GO term relationships: A: GO_1 _is a "parent" of GO_2 _in a single path relationship. B: GO_1 _is a "parent" of GO_2 _in a multiple path relationship. C: GO_1 _is a "child" of GO_2 _in a single path relationship. D: GO_1 _is a "child" of GO_2 _in a multiple path relationship. E: GO_1 _and GO_2 _are "siblings" in a single path relationship. F: GO_1 _and GO_2 _are "siblings" in a multiple path relationship. MF denotes the molecular function node (root).

**Figure 2:**
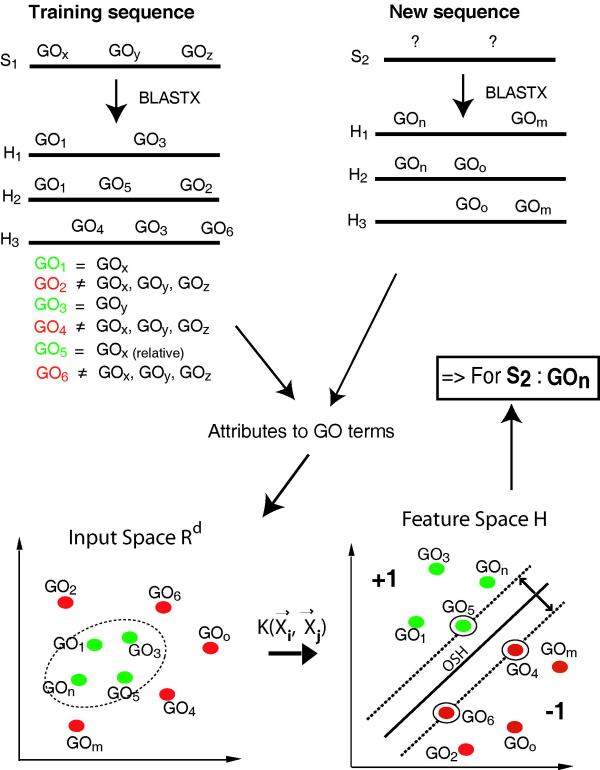
General prediction scheme: The training sequences (S1) with known function (GO_x_, GO_y_, GO_z_) were searched across the protein databases, yielding hits with molecular function GO terms (GO_1_, GO_2_, GO_3_, GO_4_, GO_5_, GO_6_) and their features (see methods), sketched as dots in a two-dimensional feature space. If GO terms of the hits compared to GO terms of the query, they were classified as +1 (correct, green), and -1 otherwise (red). The classifier (SVM) separated the classes by an optimal separating hyperplane (OSH). Unknown sequences (S2) were searched in the same manner and the GO terms (GO_n_, GO_m_, GO_o_) were extracted. Their features were calculated and mapped into the feature space. The corresponding labels were assigned (correct/false).

We selected GO-annotated cDNA sequences for training the SVM classifier. The nucleotide sequences were searched against GO-mapped protein databases and GO-annotations were extracted from the significant hits. Then, each GO term obtained was utilized as a sample for the feature table. The sample GO terms were then labelled as either correct ("+1") or false ("-1") by comparing them to the original annotation. Note that we applied the relationships of the GO terms based on their graph structure: "Correct" was assigned not only if they were exact matches but also if the GO terms were related as either "parent" or "child" (Figure 1). Next, the samples were attached with their features or attributes, calculated from the BLAST [27] results. With this data, the classifier was trained to distinguish between the attribute patterns that contributed to class +1 (correct prediction of a GO term) and class-1 (false prediction). To predict the function of unknown sequences, the same procedure was applied as for the training sequences in order to obtain their GO terms and corresponding attribute values. According to these attribute values, the classifier assigned a class for every GO term of the BLAST hits (Figure 2).

### Datasets for training and testing SVM

For training and testing the SVM, we selected 39,740 GO-annotated cDNA sequences from the following organisms: *Saccharomyces cerevisiae *(yeast), *Drosophila melanogaster *(fly), *Mus musculus *(mouse), *Arabidopsis thaliana *(*Arabidopsis*), *Caenorhabditis elegans *(worm), *Rattus norvegicus *(rat), *Danio rerio *(fish), *Leishmania major *(*Leishmania*), *Bacillus anthracis Ame *(*Bacillus*), *Coxiella burnetii RSA 493 *(*Coxiella*), *Shewanella oneidensis MR-1 *(*Shewanella*), *Vibrio cholerae *(*Vibrio*) and *Plasmodium falciparum *(*Plasmodium*) (Table 1). From these, 55.3% of the cDNA sequences were contributed by *Arabidopsis*, mouse and fly (22.1%, 18%, and 15.2% respectively). Prokaryotic bacteria (*Bacillus*, *Coxiella*, *Shewanella *and *Vibrio*) contributed 20.6% and the remaining 24.1% of the sequences came from rat, fish, worm, *Plasmodium*, *Leishmania *and yeast. Yeast and fly are purely manually annotated datasets. Where as *Bacillus*, *Coxiella*, *Vibrio*, *Shewanella*, *Leishmania *and *Plasmodium *are mostly manually, and the rest mostly automatically annotated datasets. Manual annotation tends to be conservative and sparse, since the GO terms are assigned only if the annotator is highly confident. Therefore, a GO term may be missed due to a poor definition of a false negative. To reduce this critical problems, yeast and fly annotations are accompanied by an "unknown molecular function" term for sequences with questionable further functions. To reduce false negatives, we discarded all sequences with these tags for training and testing (yeast: 2999 discarded out of 6355, fly: 8495 out of 14335).

The cDNA sequences were searched across the protein databases covering a wide range of organisms from prokaryotes to eukaryotes and SWISSPROT. For 36,771 sequences we got hits with GO terms, contributing to 856,632 sample GO terms and yielding an average of 23.29 GO terms per query sequence (Table 1). These 856,632 samples were used to train our classifier. Generally, the number of GO terms per sequence was less for prokaryotes than for eukaryotes. Rat had the maximum number of GO terms per sequence (36.9), followed by fish (32.1) and worm (27.13). In contrast, *Shewanella*, *Coxiella *and *Vibrio *sequences had the lowest number of GO terms per sequence (10.78, 12.33 and 12.54, respectively).

### SVM training and testing

#### SVM training

We set up multiple classifiers by splitting the whole dataset (856,632 samples) into 99 equal subsets. Note that, amongst these 99 subsets, 96 contained data from a single organism and the remaining 3 from two organisms each. Subsequently, we built 99 classifiers with these subsets. Since the training sets were created organism-wise, the classifiers were trained from different ranges of data, based on purely manual annotation (yeast, fly), mostly automated annotation or a mixture of both. For training each of these classifiers, we performed a model selection (parameter optimisation by cross-validation; see Methods), which yielded varying accuracy values ranging from 78.81% to 96.03%, with an average accuracy of 85.11%.

#### SVM testing

To test the classifiers performance, we prepared 13 test sets (each set corresponding to a single organism) using the same 856,632 sample GO terms. The prediction quality of all 99 classifiers were assessed by an organism-wise cross-validation approach, i.e. for each organism (test set), we used all the classifiers for prediction except those that corresponded to the same organism. With this approach, we were able to simulate the annotation of a new organism. The number of classifiers used for predictions varied highly across organisms (maximum: *Plasmodium *and *Leishmania*, 98 classifiers; minimum: *Arabidopsis*, 74 classifiers). The quality of the predictions was estimated by comparing the predicted terms with the original annotation and the results were expressed in terms of *precision *and *accuracy *values (see Methods). The average-accuracy refers to the average of the accuracy values attained by all classifiers used for the prediction. The maximum average-accuracy was achieved for fly (81.51%), followed by yeast (80.50%), and the minimum for mouse (76.0%).

Additionally, we compared the classification efficiency of the classifier derived from automatic annotation (mouse, worm and *Arabidopsis*) with the manually annotated test sequences (yeast and fly). The prediction of the yeast and fly sequences with the 20 classifiers from the mouse sequences produced an average-accuracy of 79% and 80% respectively. Similar results were acquired with the 25 classifiers from *Arabidopsis *(79% and 80%). Likewise, the worm classifiers (11 classifiers) yielded the average-accuracy of 82% for yeast and 83% for fly. These values were comparable with the average-accuracy of 81% achieved by both, using yeast as test sequences against fly classifiers (16 classifier) and vice-versa (fly test sequences against yeast classifiers). Likewise, we classified the mouse test sequences against yeast classifiers (5 classifier) and fly classifiers yielding 69% and 71% average-accuracy respectively.

### Combining multiple classification results by the committee approach

**Figure 3:**
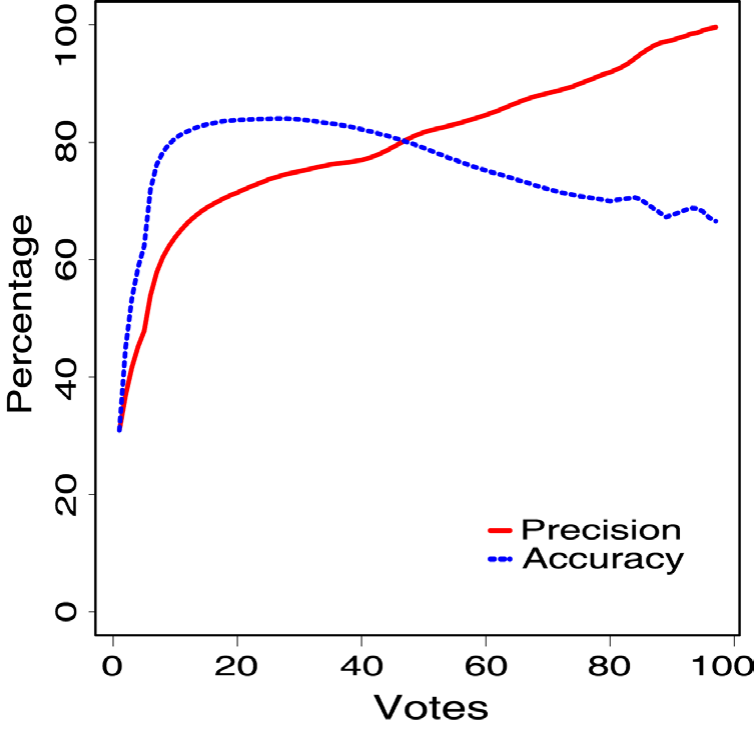
Accuracy and precision against the number of votes: The accuracy and precision values of the test data is plotted against the number of votes. An increasing number of votes increased the precision monotonically. Higher stringency yielded a sparse lowering of the accuracy due to the rate of false negatives. The relation between the precision and the number of votes was used for assigning confidence values for new predictions.

Though we already achieved a good accuracy with some of the classifiers, our intention was to improve the precision and, furthermore, to obtain confidence values for the predicted GO terms. To this end, we combined the predictions of multiple classifiers by the committee approach. If a classifier predicted a particular GO term as correct, it contributed a vote. Votes were collected from all classifiers and summed up to yield a final score value. If no vote supported a GO term as correct, it was assigned with the label "false". Otherwise, the number of votes provided a measure of the reliability. Figure 3 shows precision and accuracy versus the number of votes. If we made predictions with a minimum of one vote, we were able to achieve 43% precision and 59% accuracy. When the stringency was raised to 25 votes, a minimum of 25 votes was required to classify a GO term as correct, yielding an accuracy of 84% and precision of 75%. At a cut-off value of 74 votes, we attained 91% precision and 71% accuracy. A cut-off value of 94 votes gave 100% precision and 67% accuracy. Our accuracy reached a plateau at 20 votes. However, it decreased slightly for stringencies of more than 30 votes. Note, that this was due to the increasing number of false negatives. The relation between the precision and the number of votes (Figure 3) was used as a means of calibrating to assign the confidence values for new predictions.

**Figure 4:**
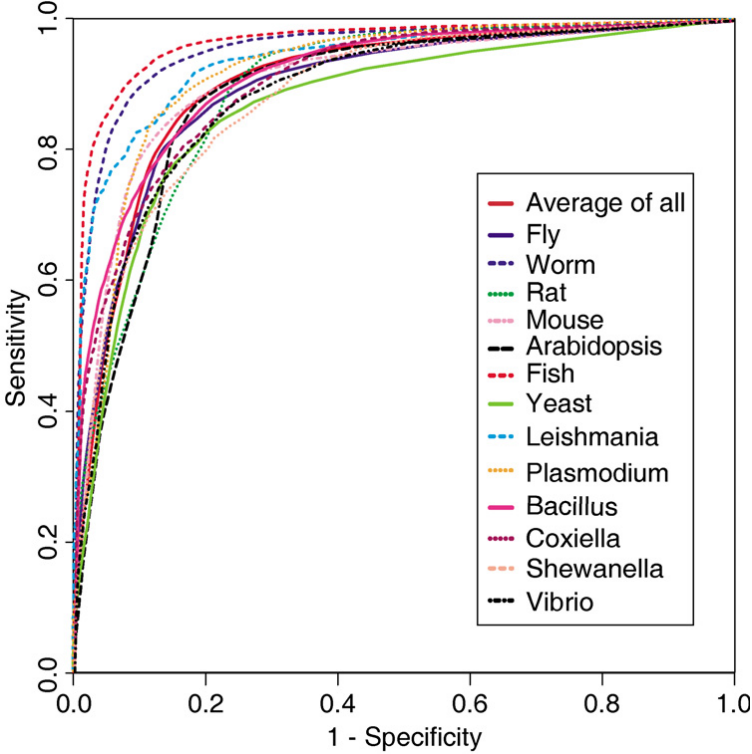
ROC plots for the classifiers performance: ROC plots for the results of all organisms tested and the average of all test sequences. The classification performance for different classes of organisms like multi-cellular eukaryotes, single-cell eukaroyotes and the prokaryotes were compared.

For each threshold value of the votes, we calculated the sensitivity and the false positive rate to obtain a Receiver Operating Characteristic plot (ROC; Figure 4). The graph shows that the classification performance was comparable for different classes of organisms like prokaryotes, single cell eukaryotes and multi-cellular eukaryotes, which reflect the organism-independent performance of our method. Note that for fish, worm, *Plasmodium *and *Leishmania *the classification performance was particularly good due to the low number but well characterised test sequences.

**Figure 5:**
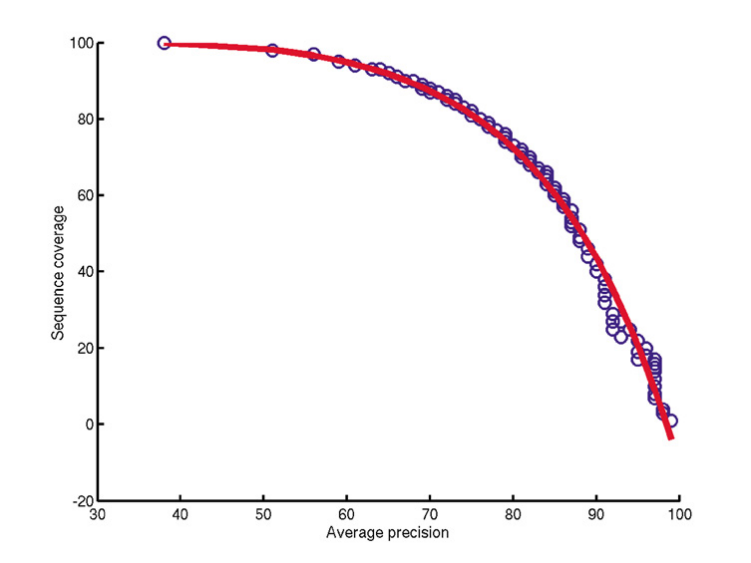
Precision against the sequence coverage: Average precision against sequence coverage for all 13-test organisms (circles). The red line denotes a fitting curve.

We compared the prediction performance for GO terms annotated with the evidence code IEA (automated annotation) and non-IEA (manually verified annotation). All sequences from *Bacillus*, *Coxiella*, *Vibrio*, *Shewanella*, yeast, *Leishmania*, and *Plasmodium *were non-IEA annotated and 99.5% of the fly GO terms were non-IEA annotated. In contrast, all sequences from fish and worm were IEA annotated. The remaining test organisms were mostly IEA annotated (rat: 88%, *Arabidopsis*: 79.4%, and mouse: 69.5%). The classification performances revealed by the ROC plots were comparable between IEA and non-IEA annotated test organisms (Figure 4). Therefore, the classifier could reproduce the annotation of other automated systems as well as high-quality manual annotation. We were interested in the coverage of sequences with respect to the average precision of the annotations (shown in Figure 5). Considering 1 vote as the cut-off value, we obtained 52% average precision for 98% coverage. We obtained 80% average precision for 74% coverage (cut-off: 34 votes), and 90% average precision for 42% coverage (cut-off: 65 votes). These coverage values varied when regarding the test organisms individually. The coverage for different test organisms at 80% average precision were: fish 97%, *Coxiella *89%, worm 88%, *Vibrio *86%, rat 85%, *Bacillus *83%, *Plasmodium *81%, mouse 78%, *Leishmania *76%, *Shewanella *74%, *Arabidopsis *69%, fly 66% and yeast 57%.

### *Xenopus *annotation

We extracted all *Xenopus laevis *contig sequences from the TIGR *Xenopus laevis *Gene Index (XGI) [28] and got a total of 35,251 contig sequences, excluding singletons. We applied our method to predict functional GO terms for these contig sequences. We predicted the function for 17,804 sequences with an average of 12.16 GO terms per sequence. In total, 23.4% of all the GO terms were predicted with less than 50% confidence value, 51.5% of them were between 50% to 80% confidence and the remaining 25% with a predicted confidence value of above 80%. At 80% stringency (predicted if the GO term possessed a confidence value of 80% or more), we made predictions for 9,510 contig sequences including 55,994 GO terms, yielding on average 5.88 GO terms per sequence.

**Figure 6:**
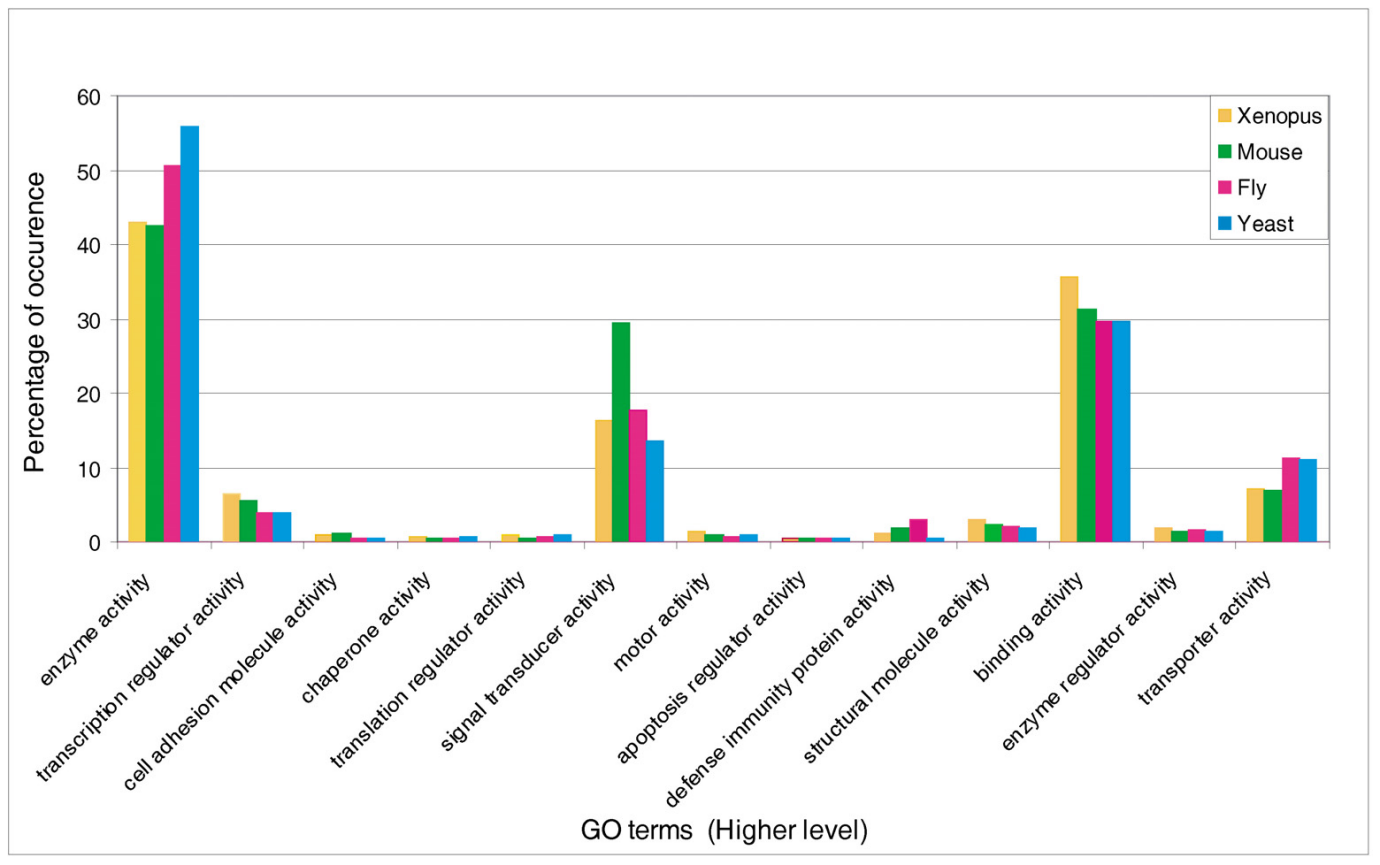
Comparison of GO slims between *Xenopus*, fly, yeast and mouse: Distributions of higher-level GO terms (,,GO slim", see text) for *Xenopus*, fly, yeast and mouse. The sum of all high-level terms may exceed the total number of the annotated terms, since some terms may have more than one high-level "parent" terms due to multiple paths.

To compare the functional abundance of the expressed genome across the organisms, we mapped the predicted GO terms (with at least one vote) to the high-level, i.e. more generalised or high-level terms of the molecular function ontology ("GO slim" for molecular function) [29]. These molecular function GO slim nodes were taken from the second level of the molecular function ontology. The distribution of higher-level GO terms were compared between *Xenopus*, fly, yeast and mouse (Figure 6). Note that some of the deeper-level terms had multiple paths. They were mapped to two or more higher-level nodes, so that the total sum of the higher-level nodes exceeded 100%.

### Comparison to the TIGR *Xenopus *annotation

TIGR provides a GO mapping for *Xenopus *contigs (TIGR *Xenopus laevis *gene indices). We compared our annotation with the TIGR GO annotation for molecular function. From 35,251 contig sequences, TIGR annotated 5,444 contigs with a total of 16,432 molecular function GO terms. In contrast, our approach was able to predict function terms for 17,804 contigs, i.e. more than three times that of TIGR sequences. Our procedure did not annotate 295 contigs from the TIGR annotated contigs. For the remaining 5,149 contigs, 85% of all TIGR terms were found to be exact with those using our method; 3.2% of the TIGR terms were at a higher-level of the GO tree than our annotation, so in this case we provided annotation at a deeper level; in 0.9% of the cases our annotation was at a higher-level; 8.3% of the cases were completely different; and 0.6% of the TIGR terms were obsolete. We compared the quality of TIGR and that of our annotations by a raising stringency and found that when we applied a confidence threshold of 80% for our annotation, we lost 46.6% of the sequences. This included 1,492 sequences holding equivalent TIGR annotation or 27.4% of the total TIGR annotation. With this stringency, our system annotated 9,510 contig sequences, i.e. twice the TIGR annotation at this quality.

We were interested in novel annotated sequences with the highest confidence values and found we could predict GO terms for 557 contigs with a confidence value of 100% (all votes matched). Interestingly, 192 of these lacked any GO annotation by TIGR. Out of these, 184 had got a descriptive TIGR annotation and the rest had not got any. Table 2 shows the novel annotation for these eight sequences. Our novel predictions are as follows: 1) TC212171 and TC196381 are predicted to display *endopeptidase activity *and more specifically *serine-type peptidase activity *(98% and 97% confidence respectively). 2) TC209487 and TC190605 are predicted to be *aminopeptidases, *however for the latter the more specific prediction of *prolyl aminopeptidase activity *is assigned with 86% confidence. 3) TC199713 is predicted as *glutathione peroxidase *at 100% confidence and TC194305 is annotated as *protein kinase *with the same confidence. 4) Both TC187949 and TC210151 are *transmembrane receptors *but the latter one is classified as *frizzled receptor *with 82% confidence. In most of these examples the functional assignment and associated confidence were recorded in multiple levels of granularity.

## Discussion

In this paper, we presented an automatic annotation system that is able to cope with the expanding amount of biological sequence data. Our approach efficiently combines the ongoing efforts of Gene Ontology and the availability of GO-mapped sequences with a profound machine learning system. The GO-mapped databases provide annotation described in a controlled vocabulary and also a measure of reliability, as these GO entries are labelled with their type of origin. Furthermore, GO terms are structured hierarchically, which allow us a twofold use of the information: i) the level within the tree is taken as a classification criterion to distinguish low from high-level annotations during the learning procedure, and, ii) the hierarchical structure allows us to extend hits by slightly moving up and down within a restricted local area of the tree. This may overcome fluctuations of the annotation levels coming from varying annotation experts.

Our annotation system exploits the different combinations of attributes and yields functional transitivity: SVM learning and prediction are organism-independent and comparable to manual annotation, which may be supported by the nature of the attributes we utilise. Subsets and overlaps are counted in a balanced fashion to avoid biases due to the complexity of an organism and a potentially correlated complexity of its sequences. The committee approach allows us to improve the prediction quality as well as to assign confidence values for the new predictions in a straightforward manner. Our classifiers performance is hardly limited by the varying quality of the training data, whether manual or automatic annotated. The prediction results of manually annotated test sets with the classifiers based on automated annotation as well as classifiers based on manual annotation were comparable. Regarding the outcome of the overall classifiers, we achieve consistency with existing annotation from automatic annotations. This is the less complex part of our work and shows a comparable efficiency of our system. Additionally, our system reproduces annotation of purely manually annotated datasets (fly, yeast, etc). However, the performance results for these datasets are low in terms of recall, i.e. 47.4% recall with 80% precision compared to 60.6% recall with the same precision of the complete test set. Note that manual annotation tends to be conservative and sparse, yielding stringent true positive definitions, whereas automatically annotated sequences may accumulate information to a greater extent.

We were interested in annotating *Xenopus *since it is a familiar model organism. However, the sequences were not very well annotated. Our system was applied to annotate the *Xenopus *contig sequences from TIGR. Through our approach, we annotated 50.5% of all contig sequences available at present, and associated a confidence value for each prediction, yielding roughly three times more sequences as compared to the currently available GO annotation. However, the coverage of annotation to new organism like *Xenopus *is crucial. We were able to attain predictions for 50.5% of all *Xenopus *contig sequences (no singletons). This compares to the applied databases that contained 53% satisfactory annotation for their sequences (not regarding sequences with unknown function terms), and better than the organism specific databases (36%). Obviously, improving the quality and quantity of annotation within the available databases goes along with the coverage exploit of machine learning algorithms for new organisms. In future we want to extend our method with the information from other sources such as domain databases and protein family databases.

## Conclusions

We developed an automated annotation system to assign functional GO terms to an unknown sequence. We used the well-established technique of Support Vector Machines (SVM) for the classification of correct and incorrect GO terms. Our approach benefited from the broad variety of potential attributes used for the functional transitivity and a vast amount of data used for training and validating. The committee scheme exploited in our system provided a means to assign confidence values in a straightforward manner. Our system performance was robust, organism-independent and reproduced the high-quality manual annotation. When applying it to *Xenopus laevis *contig sequences, we obtained a remarkably enhanced annotation coverage compared to the existing annotation.

## Methods

### Quality criteria for assessing the performance of the classifier

We used the following statistical terms [30,31].

*Accuracy *was the rate of correct predictions compared to all predictions,

Accuracy: = (TP + TN) / (TP + FP + TN + FN),     (1)

where TP denotes true positives, FP false positives, TN true negatives and FN false negatives. *Precision *was the portion of true positives with respect to all positives,

Precision: = TP / (TP + FP).     (2)

Also used were *sensitivity *:= TP / (TP + FN), *specificity *:= TN / (FP + TN), and *false positive rate *:= 1 - specificity. We defined the term *"coverage-of-sequences" *as the portion of query sequences for which the classifier delivers a prediction; "*Precision-per-sequence" *the (average) portion of correct GO terms for a single query sequence, with respect to all GO terms assigned to it. Note that these terms were defined within our model, i.e. a good "accuracy" meant good consistency with respect to our training and test sets.

### Defining the GO term relationships

We focused on the molecular function terms from GO, because the information extracted from the gene products is usually more predictive for determining molecular functions than for biological processes or cellular components. The functional terms and their hierarchy were obtained from the web pages of the Gene Ontology Consortium [29] (version of June 2003). In our study, relationships "is-a" and "part-of" were not distinguished. Note, that the "part-of" relationship is rare in the molecular function ontology (26 out of 6521 child-parent relationships). The annotation level varies across databases depending on the curator's individual knowledge about the gene product. To consider varying levels of annotation in the databases for similar gene products, we traced the relationships to match GO terms of different granularity for the same function. To find a relationship between two terms, the whole path of a GO term was traced back to the root (the root is the "molecular function" node, GO:0003674). We defined the distance between two GO terms as the distance of the shortest path. GO terms are organised in directed acyclic graphs, i.e. a child (more specialised term) may have multiple parents (less specialised terms). Therefore, we defined single path and multiple path relationships. In the case of single path relationships, GO terms had only one possible path to the root. The relationship of the term GO_1 _with respect to GO_2 _was classified as "parent", "child", "sibling" or "different" (Figure 1) according to the following rules:

GO_2 _is a *"parent" *of GO_1 _if their respective paths P_2 _and P_1 _intersect in such a manner that

P_1 _⊂ P_2_,     (3)

P_*i *_denotes the set of nodes from GO_*i *_to the root

GO_2 _is a *"child" *of GO_1 _if their paths P_2 _and P_1 _intersect such that

P_1 _⊃ P_2_,     (4)

GO_2 _is a "*sibling" *of GO_1 _if a common parent exists with a distance of one to GO_1 _and GO_2 _(Figure 1E). To avoid ambiguities for less differentiated terms, the sibling relationship was set only, if GO_1 _and GO_2 _were at least 5 nodes away from the root.

The relationship *"different" *was set if none of the previously stated criteria was fulfilled.

We could apply the single path relationship for most of the GO terms (3665 out of 5391). However, for the remaining 1726 terms more than one path to the root were found. For these cases we defined multiple path relationships and each path was considered individually. The single path relationship was applied to each possible pair of these paths (path for GO_1 _and GO_2_, respectively) and is henceforth referred to as "path-pairs". This method could yield a list of several relations. To select the appropriate relation from this list, we considered the parent relationship to be most relevant, followed by the child relationship, and the sibling was considered least relevant. We implemented the following order:

1. The parent relationship was set if at least one of the path-pairs gave a (single path) parent relationship;

2. The child relationship was set if at least one of the path-pairs gave a child relationship. To avoid a bias due to an overwhelming number of path-pairs that did not match, we set a threshold: we considered this relationship only, if the number of path-pairs with no child relationship was equal or less than four times the number of path-pairs with child relationship;

3. The sibling relationship was set if at least one of the path-pairs gave a sibling relationship. We again set a threshold: we considered this relationship only, if the number of path-pairs with no sibling relationship was equal or less than twice the number of pairs with sibling relationship;

4. If none of these criteria could be applied, the relationship "different" was set.

Note that we also implemented the hierarchy of these relations by tuning the stringencies for the fractions of path-pairs that must match (parent: no threshold, child: 1/4, sibling: 1/2).

### Data basis used for this study

Since the function transitivity at the protein level is more reliable, we used GO-mapped protein databases for searching homologues. Gene association files were obtained via the Gene Ontology Consortium. By combining the gene association files with corresponding sequence databases we created the unified protein databases. The following organisms were used: yeast, fly, mouse, *Arabidopsis*, worm, rat, fish, *Leishmania*, *Bacillus*, *Coxiella*, *Shewanella*, *Vibrio*, *Plasmodium*, *Oryza sativa*, *Trypanosoma brucei*, and *Homo sapiens*. Apart from this, the SWISS-PROT database was also included [32]. For SVM training and testing we selected 39,740 cDNA sequences from 13 organisms. These cDNA sequences were collected from the following organisms: yeast, fly, mouse, *Arabidopsis*, worm, rat, fish, *Leishmania*, *Bacillus*, *Coxiella*, *Shewanella*, *Vibrio *and *Plasmodium *(see Table 1). Out of all the known cDNA sequences we extracted 39,740 with GO molecular function terms, discarding incompletely annotated ones, i.e. sequences assigned with the GO term "molecular function unknown" (GO:0005554).

### Computing the attributes

Each cDNA sequence was searched across the protein databases, using BLASTX within the HUSAR system [33]. A query sequence was not searched within the database of their own organism. In case of SWISSPROT, hits corresponding to the query (cDNA) organism were filtered out. The BLAST files were parsed using the BLAST parser modules from W3H [34] and a low-stringent e-value cut-off of 0.01 was applied to yield a high number of possible hits. Multiple high scoring segment pairs were combined as described elsewhere [35] and used for computing the alignment features. GO terms for each database hit were extracted by considering only GO terms corresponding to the molecular function and by discarding GO terms that were prefixed with NOT (annotators state that a particular gene product is NOT associated with a particular GO term), or corresponding to "molecular function unknown" (GO:0005554). These steps reduced our dataset to 36,771 sequences, contributing to 856,632 samples. Each GO term that occurred in the hits represented a sample entry in the feature table. Below it will be referred to as "sample GO term". If a GO term occurred several times in the hits, it was considered only once. We defined 31 attributes for each GO term, representing 5 major classes of information (A)- E)):

A) GO level and path: The GO structure was exploited to derive the first two attributes,

A.1.*GO level*: the distance of the sample GO term to the root (molecular function node);

A.2. *GO path*: the number of paths from the sample GO term to the root.

B) Alignment quality criteria: These attributes are based on the BLAST alignments. For attributes B.1 - B.4, the best value for the corresponding attribute was taken, if a GO term occurred in more than one hit,

B.1. *Expectation value: *the expectation value ("E-value") from BLASTX;

B.2. *Bit score*: the bit score value provided by BLASTX;

We wanted to award alignment length and quality by combining features. This was done with respect to the length of the query *and *the hits to offset biases due to different complexities of the query and subject organisms. Attributes B.3, B.4, C.3 and D.3 were obtained from initial trials with a small dataset (6270 cDNA sequences, data not shown) and applying parameter optimisation to distinguish the samples.

B.3. *Query coverage score *(QC_S_): Combined measure of alignment size and quality concerning the query sequence,

QC_S _:= (A_L _/ Q_L_) × (I + S),     (5)

where A_L _denotes the alignment length, Q_L _the length of the query sequence, I the number of identities in the alignment, and S the number of positively contributing residues in the alignment;

B.4. *Subject coverage score *(SC_S_): as in B.3, however only with respect to the corresponding subject sequence (database hit),

SC_S _:= (A_L _/ S_L_) × (I + S),     (6)

where S_L _denotes the length of the subject sequence;

Additionally, we decomposed these attributes into the following further six attributes (B.5 - B.10). For these attributes, we considered the hit with the best coverage score if a GO term occurred in more than one hit (query coverage score for attributes B.5, B.7, B.9, and subject coverage score for B.6, B.8, B.10).

B.5. *Query percentage *(QP_C_): Percentage of coverage of the alignment region in the query sequence (with respect to QC_S_), i.e.

QP_C _:= (A_L _/ Q_L_) × 100;     (7)

B.6. *Subject percentage *(SP_C_) Percentage of coverage of the alignment region in the corresponding subject sequence (with respect to SC_S_), i.e.

SP_C _:= (A_L _/ S_L_) × 100;     (8)

B.7. *Query identity *(QI): Percentage of identical residues in the BLASTX alignment (with respect to QC_S_);

B.8. *Subject identity *(SI): Percentage of identical residues in the BLASTX alignment (with respect to SC_S_);

B.9. *Query similarity *(QS): Percentage of similar or positively contributing residues in the alignment (with respect to QC_S_);

B.10.*Subject similarity *(SS): Percentage of similar or positively contributing residues in the alignment (with respect to SC_S_).

C) GO frequency related attributes: We extracted information about the frequency of GO terms in the hits by the following attributes:

C.1.*GO frequency *(F_G_): the number of hits that contained the sample GO term.

C.2.*Number of hits *(T_H_): the total number of hits for the query.

C.3. *Frequency score *(F_S_): the number of hits that contained the sample GO term. Unlike C.1, we limited this score to emphasize differences in queries with few hits:


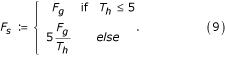


C.4.*Species frequency*: The number of organisms contributing to a sample GO term for a single query sequence;

C.5.*Total GO *(T_G_): total number of GO terms from all hits.

C.6. *Unique GO *(U_G_): as C.5, except, that GO terms occurring more than once (in the hits) were counted only once.

D) GO frequency by considering relationships: For these attributes we applied the structure of the Gene Ontology graph. Not only perfectly matching terms were considered, but also their defined parents, children or siblings:

D.1.*Relative frequency for all (R*^A^): the relationships for the sample GO term with all GO terms that occurred in the hits were calculated. The sum of non-"different" relationships i.e. parent, child, or sibling was used for this attribute;

D.2.*Relative frequency for unique *(R^U^): similar to attribute D.1, with the exception that GO terms occurring more than once were counted only once.

D.3.*Relative frequency for all (limited) (R*^*Alim*^*): *same as attribute D.1, however this score was limited to emphasize differences of queries with few hits:


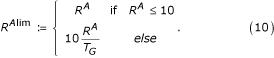


D.4.*Relative frequency for unique (limited) *(R^*Ulim*^): same as attribute D.2, however this score was limited to emphasize differences of queries with few hits:


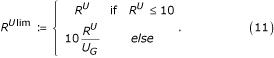


E) Annotation quality related attributes: Quality attributes were selected from the evidence codes provided by the gene association tables of the GO-mapped sequence databases. We selected 9 commonly used evidence codes (TAS, NAS, ISS, IPI, IMP, IGI, IEP, IEA, IDA), resulting in attributes E.1 to E.9. The entries of these attributes for each sample GO term were calculated by summing the occurrences of the corresponding evidence codes of all hits.

### Training and testing the classifier

Before training, normalisation was performed. We normalised the attributes by taking the logarithm (log) and log of log if necessary. We used log values for 16 attributes (B.3-B.10, C.3, C.4, D.1, D.2, D.4 and E.1) and log of log for 8 attributes (B.2, C.1, E.2, E.4-E.8). Furthermore, we converted the attribute values into mean 0 and standard deviation 1 by applying the Z-transformation. The feature table contained 856,632 samples and 31 attributes. We split the dataset into 99 training subsets. Each subset comprised of approximately 1% of the samples i.e. 8,566 GO terms. This resulted in 96 organism specific subsets and 3 hybrid subsets. We applied the support vector machines in the implementation of LIBSVM [36], which supports a weighted SVM for unbalanced data. We used a higher penalty (5 instead of 1) for false positives (FP) for the model selection and also the training process to support a high specificity of the resulting classifiers. Also note, that our training set contained a high portion of negative samples (see Table 1) due to our relaxed E-value threshold. We utilised the radial basis function kernel and set the parameter epsilon (tolerance of termination criterion) to 0.01. The parameter C (regularisation term, cost for false classification) and gamma (kernel width) of the SVM were optimised using a grid search. The grid search determined the combination of C (log2-range: 13 to 15, step 1) and gamma (log2-range: 10 to 15, step 1) with the lowest classification error according to a five-fold cross validation such that each of the 99 data subsets was subdivided into a training set (90%) and a validation set (10%). The validation sets were used to estimate the parameters C and gamma for each of the 99 classifiers individually. Finally, the parameters from the classifier selection were applied to train each of the classifiers with 90% of each data set, respectively. The testing was based on the same 13 organisms and 856,632 GO terms corresponding to 36,771 sequences as described above. We performed the testing by an organism-wise cross-validation so that one organism was used as test set and the remaining ones as the training set.

### Data availability

The annotation for *Xenopus laevis *contig sequences is downloadable at . We followed the standard GO annotation style (using Gene ontology guideline). The evidence code is always IEA. The confidence value is included for each GO term.

## Authors' contributions

The main work was carried out by AV. RK and KG conceived the idea of the study. AV and RK drafted the manuscript. FS developed and JM applied the machine learning strategy. KG implemented the databases in SRS. RE and SS supervised the work. All authors participated in reading, approving and revising the manuscript.

## Figures and Tables

**Table 1:** Training and test dataset: Datasets used for training and testing the support vector machines. The columns are: 1. The number of cDNA sequences for training. 2. The number of cDNA sequences with BLAST hits having GO molecular function terms. 3. The average number of GO molecular function terms per cDNA sequence of the BLAST-hits. 4. The classification of GO terms coming from the hits, positive if the GO terms were similar to original annotation, negative otherwise.

**Organisms**	**Number of cDNAs**	**cDNA with MF GO**	**Number of GO/cDNA**	**Class distribution**	
				**% Positive**	**% Negative**
Rat	1039	1036	36.90	25.7	74.3
Fish	1061	1044	32.10	39.2	60.8
Fly	5840	5574	25.47	23.4	76.6
Worm	4272	3458	27.13	39.5	60.5
*Plasmodium*	274	271	23.67	28.0	72.0
*Leishmania*	82	82	20.51	35.1	64.9
Yeast	3356	2972	18.60	23.7	76.3
*Bacillus*	2729	2577	13.63	35.4	64.6
*Coxiella*	931	900	12.33	37.0	63.0
*Shewanella*	2413	2303	10.78	33.0	67.0
*Vibrio*	1832	1804	12.54	31.9	68.1
*Arabidopsis*	8807	8120	26.66	30.2	69.8

**Table 2:** The novel predictions with high confidence values (100%). The columns correspond to TC (TIGR-id) number, GO term level in GO-structure, the confidence values and the GO number and GO terms.

**TC Numbers**	**GO level**	**Confidence value**	**GO ID**	**GO terms**
TC212171	4	100%	GO:0008233	peptidase activity
	5	100%	GO:0004175	endopeptidase activity
	5	98%	GO:0008236	serine-type peptidase activity
	3	98%	GO:0016787	hydrolase activity
TC196381	5	100%	GO:0004175	endopeptidase activity
	4	98%	GO:0008233	peptidase activity
	3	98%	GO:0016787	hydrolase activity
	5	97%	GO:0008236	serine-type peptidase activity
TC209487	2	100%	GO:0003824	enzyme activity
	3	100%	GO:0016787	hydrolase activity
	5	90%	GO:0004177	aminopeptidase activity
	4	85%	GO:0017171	serine hydrolase activity
TC187949	4	100%	GO:0004888	transmembrane receptor activity
	3	97%	GO:0004872	receptor activity
TC194305	2	100%	GO:0003824	enzyme activity
	3	99%	GO:0016740	transferase activity
	3	99%	GO:0016301	kinase activity
	6	97%	GO:0004672	protein kinase activity
TC210151	3	100%	GO:0004872	receptor activity
	4	97%	GO:0004888	transmembrane receptor activity
	6	82%	GO:0004928	frizzled receptor activity
TC199713	6	100%	GO:0004602	glutathione peroxidase activity
	3	98%	GO:0016491	oxidoreductase activity
	5	85%	GO:0004601	peroxidase activity
TC190605	2	100%	GO:0003824	enzyme activity
	3	100%	GO:0016787	hydrolase activity
	4	87%	GO:0017171	serine hydrolase activity
	6	86%	GO:0016804	prolyl aminopeptidase activity
